# Review of yeast culture concerning the interactions between gut microbiota and young ruminant animals

**DOI:** 10.3389/fvets.2024.1335765

**Published:** 2024-03-01

**Authors:** Shixiong Liu, Lan Yang, Yufei Zhang, Hui Chen, Xueqiang Li, Zixuan Xu, Rui Du, Xiao Li, Jiabin Ma, Dacheng Liu

**Affiliations:** College of Veterinary Medicine, Inner Mongolia Agricultural University, Hohhot, China

**Keywords:** young ruminants, weaning stress, yeast culture, intestinal immunity, intestinal flora

## Abstract

Microorganisms inhabit the gastrointestinal tract of ruminants and regulate body metabolism by maintaining intestinal health. The state of gastrointestinal health is influenced not only by the macro-level factors of optimal development and the physiological structure integrity but also by the delicate equilibrium between the intestinal flora and immune status at the micro-level. Abrupt weaning in young ruminants causes incomplete development of the intestinal tract resulting in an unstable and unformed microbiota. Abrupt weaning also induced damages to the microecological homeostasis of the intestinal tract, resulting in the intestinal infections and diseases, such as diarrhea. Recently, nutritional and functional yeast culture has been researched to tackle these problems. Herein, we summarized current known interactions between intestinal microorganisms and the body of young ruminants, then we discussed the regulatory effects of using yeast culture as a feed supplement. Yeast culture is a microecological preparation that contains yeast, enriched with yeast metabolites and other nutrient-active components, including β-glucan, mannan, digestive enzymes, amino acids, minerals, vitamins, and some other unknown growth factors. It stimulates the proliferation of intestinal mucosal epithelial cells and the reproduction of intestinal microorganisms by providing special nutrient substrates to support the intestinal function. Additionally, the β-glucan and mannan effectively stimulate intestinal mucosal immunity, promote immune response, activate macrophages, and increase acid phosphatase levels, thereby improving the body’s resistance to several disease. The incorporation of yeast culture into young ruminants’ diet significantly alleviated the damage caused by weaning stress to the gastrointestinal tract which also acts an effective strategy to promote the balance of intestinal flora, development of intestinal tissue, and establishment of mucosal immune system. Our review provides a theoretical basis for the application of yeast culture in the diet of young ruminants.

## 1 Introduction

In intensive farming, the natural weaning time of young animals is often advanced to enhance production efficiency. However, these animals are subjected to this early weaning, when their digestive and immune systems are immature. Combined with the stress of separation from the mother (in some instances), dietary changes, altered feeding methods, and environmental shifts, the weaning induces a range of psychological and physiological stress responses in the animals. These responses can result in indigestion, stunted growth, decreased immunity (1) or more severe outcomes such as diarrhea and mortality in weaned animals. These phenomena are largely attributed to the alterations in the intestinal flora because of weaning stress (2). The animal gut hosts a myriad of microorganisms that symbiotically interact with their host, offering mutual benefits. These microorganisms not only enhance nutrient availability and food digestibility in the gut but also play a crucial role in the development of the immune system of the animal (3). This interaction accelerates the maturation of the immune system and bolsters pathogen elimination. Furthermore, the gut microbiota is reflective of co-metabolism and the symbiosis between microorganisms and the animal, significantly influencing the health and development of the animal (4). They are also recognized as a critical environmental factor that regulates immune function through metabolic exchanges.

Addressing weaning stress in lambs is a significant challenge, especially in the context of antibiotic bans in feed. Biological feed additives have shown promise as antibiotic-free alternatives. Microecological preparations not only enhance animal immune function, alleviate oxidative stress, and improve disease resistance (5) but also promote gastrointestinal floral balance and nutrient digestibility (6). For instance, yeast cultures can boost immunity and antioxidant capacity (7), modulate inflammatory factors, and exhibit other biological effects (8). Han et al. (9) highlighted the role of microecological preparations in fostering intestinal development and establishing microbial communities. Xinxu et al. (10) showed that fermented feed significantly improves the growth performance of weaned piglets, altering their intestinal microflora, serum biochemical indices, and immune indices. Additionally, introducing yeast cultures during early weaning could enhance rumen microbial colonization, improve intestinal development and digestibility, and promote lamb growth (11). However, understanding the vast and complex gut microbial network remains a challenge, with existing research being somewhat fragmented. Therefore, investigating the interactions between yeast cultures and gut microbes in young ruminants is crucial for understanding their impact on animal welfare and health.

## 2 Effects of intestinal microorganisms on young ruminants

### 2.1 Effects of intestinal microorganisms on the metabolism of young ruminants

Nicholson et al. (12) introduced the concept of the host-microbe metabolic axis, defining it as a system connecting specific host cellular pathways with a range of microbial species, sub-ecosystems, and microbial metabolic activities through multidirectional, reciprocal, high-speed chemical signals. In this metabolic axis, multiple microbial genomes collaborate to regulate metabolic processes, enabling microbial metabolites to interact with the host genome (13). Beneficial metabolites produced by microorganisms, such as bile acids, choline, and short-chain fatty acids (SCFAs) (14), contribute to the host’s health (15). Furthermore, these microbial metabolites influence the metabolic phenotype of the host, potentially reducing disease risk. The intestinal microorganisms form a host-microbial metabolic axis within the animal’s body, playing a crucial role in nutrient metabolism and immune response in the animal (16). The normal microbial flora in the gut metabolizes both externally ingested and endogenous macromolecules like carbohydrates, proteins, and fatty acids. Microorganisms also interact with the body metabolism to produce a variety of metabolites, including SCFAs, amino acids, small peptides, polyamines, bile salts, and methyl donors. These metabolites are instrumental in the metabolism of substances and stabilization of the immune system in both intestinal epithelial tissue and the entire body (12). Additionally, intestinal bacteria produce pathogen-associated pattern molecules, such as lipopolysaccharides and peptidoglycans, which can elicit an immune response from intestinal epithelial cells (IECs). Gut microorganisms and their metabolites extensively interact with host intestinal epithelial tissue and participate in the metabolism of nutrients in the host’s intestinal cavity. Changes in microbial community structure are often accompanied by alterations in the physiological functions of the intestinal environment (17), subsequently impacting the overall metabolic homeostasis of the body. Mardinoglu et al. (18) reported that the gut microbiota regulates the metabolism of glutathione and amino acids in the host body. Glutathione, a key antioxidant present in every cell, plays a vital role in various lifestyle diseases, and its depletion can trigger oxidative stress responses in the body (18).

### 2.2 Effects of intestinal microorganisms on the intestinal mucosal barrier function in young ruminants

The intestinal mucosal barrier comprises mechanical, chemical, immune, and biological barriers (19), with the mechanical and immune barriers being the most critical. The intestinal epithelial mechanical barrier is formed of epithelial cells, including structures like tight junctions, mucinous junctions, desmosomes, and interstitial junctions, among which tight junctions are pivotal in preventing epithelial cell bypass (20). Tight junction complexes consist of various transmembrane proteins, such as the claudin, occludin, and connexin family of proteins. Notably, tight junction protein 1 (ZO-1) is associated with the actin cytoskeleton. These intestinal epithelial tight junctions serve as a primary physical barrier to prevent microorganisms in the intestinal lumen from invading sterile internal organs. The regulation of intestinal permeability by probiotics has been demonstrated (21). For instance, the probiotic mixture Val #3 (*Streptococcus thermophilus*, *Bifidobacterium longum*, *B. breve*, *B. infantis*, *Lactobacillus acidophilus*, *L. plantarum*, *L. casei*, *L. bulgaricus*), a compound of eight probiotics, when administered to rats via gavage, reduced colonic epithelial permeability by increasing the gene expression and protein content of closure proteins, including ZO-1 and closure proteins 1, 3, 4, and 5, thereby mitigating sodium dextrose sulfate-induced inflammation in the rat colon (22). *In vitro* studies have revealed that Zactokc decreases the permeability of Caco-2 epithelial cells by upregulating occludin and oingulin proteins (23). Additionally, both *Escherichia coli* NISSLE1917 and *Lactobacillus plantarum* could counteract the altered permeability of epithelial cells caused by enteropathogenic *E. coli* through mechanisms such as PKC silencing and ZO-2 upregulation (24). In contrast, conditional pathogenic bacteria like *E. coli* can reduce the expression of ZO-1, claudin, and claudin-1 proteins, thereby increasing the permeability of IECs. These results underscore the significant role of gut microbes in regulating the intestinal barrier function (25).

Intestinal microbial metabolism products, such as SCFAs, polyamines, and secondary bile acids, also play a role in gut barrier function regulation (26). SCFAs can alter the permeability of tight junctions between IECs (27). Inoculating *Bifidobacterium longum,* which produces high concentrations of acetic acid, into germ-free mice was found to confer resistance to infection by the intestinal pathogen *Escherichia coli* O157:H7. This suggests that SCFAs may effectively prevent pathogen transfer from the intestinal lumen to the circulatory system by maintaining intestinal epithelial integrity (28). Furthermore, butyric acid has been shown to significantly increase oxygen consumption in colonic IECs in mice, creating a “physiological hypoxia” effect. This effect induces the synthesis and secretion of hypoxia-inducible factors by colonic IECs, thereby sustaining intestinal barrier function. Antibiotic intervention that inhibit intestinal microorganisms can significantly reduce the intestinal SCFA content and IEC oxygen consumption, deactivating hypoxia-inducible factors and consequently weakening the intestinal barrier function (29). Additionally, *in vitro* cell assays have shown that butyric acid can regulate the recombination of ZO-1 and occludin proteins in Caco-2 epithelial cells via the AMP-activated protein kinase pathway, thus enhancing the cellular barrier function in these cells (30). A study by Burger van et al. (31) on human LS174T cells revealed that appropriate concentrations of butyric acid and propanoic acid enhanced the barrier function of IECs by boosting MUC2 expression, while higher concentrations inversely regulated this function.

### 2.3 Effects of intestinal microbes on the development of the intestinal immune system in young ruminants

Intestinal microbes are crucial in establishing the early immune system. Increasing evidence suggests a higher incidence of allergies in infants delivered by Cesarean section than in those delivered vaginally (32). Additionally, gut microbial compositions differ between healthy and allergic infants (33), indicating that early microbial colonization may be a pivotal factor in stimulating immune system maturation. Intestinal microbes can promote the differentiation of immune cells through specific components, such as regulatory T and Th17 cells (34). Disturbances in intestinal flora can lead to bacterial translocation and damage to intestinal barrier function (35), subsequently affecting overall health. Research using germ-free or germ-restricted mice has highlighted that commensal microflora in the gut are significant contributors to regulating the host’s immune system and intestinal morphology. Microbial metabolites, such as SCFAs, have been shown to stimulate the proliferation of mucosal epithelial cells (36). Oral tolerance to ovalbumin was established after reconstituting the intestinal flora of germ-free neonatal mice, but this effect was absent in adult mice (37). The germ-free mice exhibited more developmental defects in their immune systems, such as shorter and fewer crypt cells, fewer TCRαβ^+^ intraepithelial lymphocytes, lower serum immunoglobulin levels, than those in the wild-type mice systems, along with an absence of induced lymphoid follicles (38). Transplanting normal mouse intestinal flora into germ-free mice could induce the development of gut-associated lymphoid tissue, such as TCRαβ^+^ intraepithelial lymphocytes (39). Further, colonization of segmented filamentous bacteria in the small intestine of germ-free mice could restore mucosal morphology and function, elevating the number of TCRαβ^+^ intraepithelial cells to levels observed in normal mice (40). Hall et al. (41) demonstrated that bacterial DNA derived from gut microbiota mediates the balance between proinflammatory Th-17 cells and regulatory T cells. Additionally, adenosine triphosphate produced by bacteria could promote Th-17 accumulation (42), and segmented filamentous bacteria could induce the development of Th-17 cells in the lamina propria (43). Thus, early intestinal microbes play a vital role in establishing the intestinal immune system, whereas later intestinal microbes are important for the homeostatic regulation of this system. The different effects of intestinal microbes on the development of the intestinal immune system in young ruminants (44–48) is presented in Table 1.

**Table 1:** Effects of intestinal microbes on the development of the intestinal immune system in young ruminants.

Animal	Conclusion	References
Calves	Intestinal microorganisms can regulate the development of intestinal mucosal immune system in calves.	(44)
Calves	Gut microbiota plays an important role in the development of mucosal immune system.	(45)
Calves	Intestinal microflora plays an important role in the development of host mucosal epithelium and immune system.	(46)
Neonatal dairy calves	Gut microbes influence the development of the gut immune system.	(47)
Neonatal ruminants	The colonization of intestinal microorganisms has an important impact on the development of host innate immunity.	(48)

### 2.4 Effects of intestinal microorganisms on the immune response of intestinal mucosa in young ruminants

The intestinal mucosa is the initial contact point of the intestinal microorganisms with the host (49); the microbes are separated from the host’s immune system by only a single epithelial layer. Besides epithelial cells, specialized cells such as M, PAN, cup, and dendritic cells could extend their dendrites to directly sense the contents of the intestinal lumen and interact with intestinal bacteria. Upon pathogen detection, epithelial cells secrete humoral mediators like immunoglobulin A, antimicrobial peptides, chemokines, or cytokines, which activate innate and adaptive immune responses. Additionally, M cells can transfer antigens to antigen-presenting cells such as dendritic cells or macrophages. These cells then carry bacterial antigens to induce adaptive immune responses in Pan’s node cells or mesenteric lymph node cells (50).

The intestinal immune system typically has a pro-inflammatory response to pathogenic bacteria, whereas commensal bacteria often evade such responses. Although the exact mechanism is not fully understood, some strategies are known to be used by commensal bacteria to avoid the host immune system (51). For instance, commensal bacteria can inhibit NF-κB pro-inflammatory responses and the production of pro-inflammatory cytokines by mimicking host cell membrane proteins (52). Dysbiosis, or changes in the intestinal flora, has been linked to increased susceptibility to inflammatory bowel disease (IBD). Antibiotic treatments and probiotic administration have shown efficacy in improving IBD (53). Interestingly, germ-free mice neither develop IBD nor exhibit exacerbated IBD symptoms compared to those by wild-type mice (54), highlighting the crucial role of gut microbes in maintaining intestinal immunity and health.

Gut microbial metabolites, particularly SCFAs, are vital in regulating intestinal immunity (55). SCFAs, produced by anaerobic microorganisms in the large intestine during the fermentation of undigested carbohydrates from the small intestine, include acetic acid, propionic acid, and butyric acid. These acids mediate the growth, metabolism, and immune response of IECs. SCFAs are recognized by free fatty acid receptors on the surface of IECs, activating inflammation-related signaling pathways and playing a significant role in regulating the intestinal immune response. Kim et al. (56) found that SCFAs could significantly increase the expression of inflammatory cytokines (IL-1β, IL-6, and TNF-α) and chemokines (CXCL1 and CXCL2) in the mouse intestine. SCFAs also regulate intestinal innate immune responses by activating the expression of Toll-like receptors (57). Research on colonic epithelial cell lines has shown that propionic acid and butyric acid increase TLR5 expression on IECs, activating the NF-κB signaling pathway and upregulating the inflammatory cytokine TNF-α, while downregulating IL-8 and monocyte chemokine 1 (58). These findings demonstrate that SCFAs can regulate cytokine expression in intestinal IECs through the TLR5-NF-κB signaling pathway, mediating the innate immune response. Additionally, SCFAs play a role in the adaptive immune response of intestinal immune cells (59). Roman et al. (60) discovered that butyric acid could activate the FFAR3 receptor on the surface of mouse colon cells, enhancing the immune tolerance of regulatory T cells in the colon, thereby mitigating cytokine expression increases caused by colonic inflammation.

## 3 Effects of weaning stress on the health of young ruminants

The sudden separation of ewes and lambs prior to weaning can induce stress in lambs (61). The primary stressors identified include (1) emotional distress due to the separation from the mother; (2) environmental changes, as lambs are typically relocated to new surroundings; (3) cessation of lactation, stemming from neurophysiological shifts induced by lactation; and (4) nutritional transitions, occasioned by replacing milk with solid feeds. Post-weaning, both ewes and lambs exhibit increased frequencies of behaviors indicative of mutual search, such as calling, standing, walking, and pacing. Damian et al. (62) conducted a comparative analysis of stress responses in lambs at weaning. When lambs were artificially fed with ewe’s milk through artificial nipples, there was a notable increase in pacing, walking, and vocalization, alongside a significant reduction in grazing time. Early weaning also triggers changes in physiological stress indicators in both ewes and lambs (63), such as a rapid surge in serum cortisol levels. Cortisol, a primary endocrine indicator of stress response in sheep, is closely linked with the regulation of the immune response. Infections with *Haemonchus contortus* and *Trichostrongylus colubriformis* were found to be more prevalent in weaned lambs; these lambs also exhibited reduced antibody production compared to that in unweaned lambs. Moreover, weaning not only adversely impacts lamb growth but can also impede the normal development of the rumen, particularly when lambs are not artificially fed. Moreover, the dietary shift from liquid to solid feed alters the microbial flora in young ruminants, subsequently affecting their growth performance and overall health.

### 3.1 Effects of weaning stress on intestinal development and flora in young ruminants

The animal gut is a habitat for a vast array of microorganisms (64), which are pivotal not only for the growth and development of the animals but also for maintaining the dynamic balance of the intestinal system. Compared to that exhibited by their non-weaned counterparts of the same age, 28-day-old weaned lambs exhibited an increase in rumen microbial community richness. However, there were no significant changes in diversity and no significant differences in microbial community composition at the phylum level by 42 days of age (65). Yang et al. (66) discovered that early supplementation with alfalfa could align the rumen microbiota of pre-weaning lambs closer to the post-weaning composition, thereby reducing genus-level flora changes, enhancing rumen microbiota stability, and mitigating the weaning stress response. Mechanistically, previous research has demonstrated that weaning disrupts the intestinal barrier function in young animals, marked by increased permeability of the intestinal epithelium (67). Concurrently, a disruption in epithelial barrier function and the upregulation of proinflammatory cytokines lead to a pronounced activation of the gastrointestinal immune system post-weaning. The hypothalamic pituitary adrenal axis becomes activated, and stress-related mediators, such as cortisol, are elevated in young animals. This activation of the hypothalamic–pituitary adrenal axis is a crucial mechanism for coping with stressors and reestablishing homeostasis. Post-weaning, serum cortisol levels rise, and at the intestinal level, weaning stress disrupts gut microbiota homeostasis, creating favorable conditions for pathogen proliferation, which in turn leads to increased morbidity and mortality among young animals. The effects of weaning stress on the intestinal development and flora in young ruminants (1, 68–72) are summarized in Table 2.

**Table 2:** Effects of weaning stress on intestinal development and flora in young ruminants.

Animal	Conclusion	References
Lambs	Weaning stress can cause changes in the immune system of lambs.	(1)
Lambs	Early weaning significantly increases the bacterial diversity in the ileum of lambs. Simultaneously, it increases the expression of TLRs and tight junction protein genes.	(68)
Lambs	Early weaning leads to decreased growth performance and immunity of lambs, impaired intestinal morphology, and disrupted intestinal microecological balance.	(69)
Goats	Early weaning can cause intestinal damage, leading to a series of long-term symptoms such as inflammation, malabsorption and diarrhea.	(70)
Holstein Calves	Weaning stress can reduce the feed intake of calves, destroy the small intestine structure of calves, and affect their production capacity in adulthood.	(71)
Young Goats	Mammalian weaning can lead to intestinal dysfunction and intestinal microbial disorders.	(72)

### 3.2 Effects of weaning stress on the intestinal barrier function in young ruminants

Weaning marks a critical stage in the growth of lambs, characterized by a transition from breast milk to plant-based feed, with carbohydrates supplanting fat as the primary energy source (73). This enforced dietary shift and separation from the ewe constitute a significant stressor for lambs, potentially hindering their growth, development, and health. During the weaning period, issues such as reduced feed intake, weight loss, and increased morbidity and mortality are common (74), with the effects of weaning stress typically manifesting within 1–3 days post-weaning. Research indicates that early weaning escalates the permeability of the intestinal mucosa in calves (75), allowing bacteria, bacterial toxins, allergens, and other harmful agents to more easily penetrate the intestinal barrier, potentially triggering inflammation or immune responses. The gut-associated lymphoid tissue is a crucial component of the gut barrier. Because of its functional significance and extensive contact with antigens, the gut-associated lymphoid tissue plays a vital role in the immune system.

Early weaning influences the innate immune responses in calves (76). The age of weaning significantly impacts the distribution of leukocytes, cytokines, and acute phase proteins in the blood of calves treated with lipopolysaccharide, with calf blood neutrophils and interleukin-8 serving as potential biomarkers of weaning stress (77). As a key part of the body’s immune system, damage to the intestinal immune barrier induced by weaning stress can heighten susceptibility to diseases. Research has identified stress as a primary contributor to the clinical onset of conditions like irritable bowel syndrome, IBD, and chronic intestinal infections (78, 79). Post-weaning diarrhea is not only a prevalent issue in young animals, typically occurring between 3- and 10-days post-weaning, but it is also a major cause of mortality during the weaning period. Post-weaning diarrhea adversely affects post-weaning weight gain and long-term production performance (80). While there are limited reports on the effects of early weaning stress on the intestinal barrier of lambs, studies on calves and piglets have documented significant impacts of early weaning stress on intestinal barrier function. Consequently, it is hypothesized that early weaning stress may similarly affect the intestinal barrier function of lambs. Undertaking experimental studies to definitively ascertain the effects of weaning age on the intestinal barrier function of lambs is a pressing issue in the rational application of early weaning techniques.

## 4 Effects of yeast cultures on the intestinal health of young ruminants

### 4.1 Effects of yeast cultures on the intestinal morphology of young ruminants

The small intestine is a critical site for nutrient digestion and absorption in ruminants. Within this, the small intestinal villi play a pivotal role (81). The height of the small intestinal villi, the depth of the crypts, and the ratio of villus height to crypt depth (V/C value) are crucial metrics for assessing the digestive and absorptive function of the small intestine. The villus height is directly proportional to the nutrient absorption area; a decrease in height corresponds to reduced absorption capability. The small intestine crypt, a tubular gland at the base of the villi, is integral for IEC renewal. Its depth is proportional to the rate of this renewal. The V/C ratio is an overall indicator of nutrient absorption capacity in the small intestine, with a higher ratio signifying increased absorption area and functionality, and a decrease in ratio suggesting impaired digestive and absorptive capacity, negatively impacting animal growth and development (82). Park et al. (83) corroborated that yeast cell walls can safeguard the intestinal mucosa from damage. Furthermore, yeast nucleic acid contributes to intestinal development by increasing intestinal mucosal protein (84), DNA, and RNA content, enhancing the height of intestinal villi and the thickness of the intestinal wall, and boosting the activity of enzymes like maltase, lactase, and sucrase in the intestinal mucosa. In addition, yeast β-dextran can positively influence the intestinal development of weaned calves (85). The addition of yeast β-glucan can increase the height of intestinal villi in the duodenum, jejunum, and middle ileum. In calves supplemented with 75 mg/kg of yeast β-glucan, the crypt depth was significantly lower than in other groups, and the V/C value was significantly higher than that of the control group. Moreover, the thickness of the small intestine mucosa in this group was the highest among all experimental groups (86).

### 4.2 Effects of yeast cultures on the intestinal flora of young ruminants

The intestinal flora in the digestive tract of host animals is in a state of dynamic equilibrium, with bacterial populations varying in response to changes in diet, environment, and other factors. Approximately 99% of beneficial microorganisms in the animal gut are anaerobic bacteria, while aerobic and facultative anaerobic bacteria are less prevalent (87). Certain microecological agents, which are aerobic, upon entering the small intestine, rapidly revive and consume free oxygen, creating a hypoxic environment. This shift promotes the growth and reproduction of anaerobic beneficial bacteria (88) such as *Bifidobacterium* and lactic acid bacteria, while inhibiting the invasion and colonization of aerobic pathogenic bacteria like *E. coli*, thus preventing intestinal inflammation, diarrhea, and other diseases in animals. Secondary metabolites from some microecological agents can inhibit the growth of intestinal pathogens such as *E. coli* and *Clostridium perfringens*. Additionally, the SCFAs produced during their metabolic process can create an acidic environment in the intestine, fostering the growth of beneficial bacteria such as *Lactobacillus*, thereby maintaining intestinal health and enhancing production performance (89). Yeast culture is known to shorten the lag phase, prolong the logarithmic phase, and significantly increase the number of flora by promoting the proliferation of beneficial intestinal bacteria like *Bifidobacterium* and *Lactobacillus*. The regulatory effect of yeast culture on animal intestinal flora is evidenced not only in the proliferation of beneficial intestinal flora (90) but also in inhibiting harmful bacteria, such as *E. coli* through the production of enterotoxins (91). The antibacterial effect of yeast culture partly stems from the alteration of the nutrient composition of the flora. Additionally, mannan oligosaccharides and β-glucan in the yeast cell wall exert bacteriostatic effects (92). Zhaoxiaojing et al. (92) indicated that adding 0.1% mannan oligosaccharide to the feed could reduce the number of *E. coli* in calf feces and increase *Lactobacillus* levels. Mannan oligosaccharides function by competitively binding with exogenous lectins on the surface of pathogenic bacteria, preventing these pathogens from adhering to receptors on the intestinal mucosa, thereby inhibiting pathogenic bacteria.

### 4.3 Effects of yeast culture on diarrhea in young ruminants

Young animals, with their underdeveloped intestines and limited stress resistance, are prone to diarrhea, which can lead to malnutrition or even death. Supplementing their diet with yeast cultures has shown effectiveness in alleviating this issue. Brewer et al. (93) demonstrated that incorporating yeast culture into calf diets could significantly reduce the incidence of diarrhea. Magalhaes et al. (94) found that adding yeast to calf diets not only improved feces consistency, but also decreased the duration of watery feces and fever, reduced diarrhea rates, and lowered the incidence of disease in calves the first 13 days after birth, thereby improving survival rates. Additionally, Ozsoy et al. (95) indicated that adding 4.5% active yeast culture to the diet of fattening goats increased their body weight gain and reduced the total number of coliform bacteria present. Similar studies have also found that *Saccharomyces cerevisiae* fermentation products can be used to reduce diarrhea in growing calves (96).

### 4.4 Effects of yeast culture on the production performance and digestion and absorption capacity of young ruminants

With the rapid advancement of the farming industry, numerous innovative products, including enzyme preparations, herbal medicine preparations, acidifiers, and microecological preparations, have emerged to reduce costs and increase efficiency. Among these, microecological preparations, particularly yeast cultures, have demonstrated positive effects. Zhang et al. (97) revealed that adding yeast culture to the diet of beef cattle not only promotes their growth and development but also enhances nutrient digestibility and average daily gain. Maamouri et al. (98) supplemented yeast culture in the diet of weaned Holstein calves, leading to improved dry matter intake and average daily gain. This addition also beneficially affected cellulase activity and volatile acid concentration in the rumen fluid, thereby enhancing nutrient digestion and absorption rates in the calves. Another study by the same group showed that including yeast culture in the diet of fattening cattle improved their growth performance and feed digestibility (99). Supplementing total mixed rations with yeast culture products can improve the growth performance of lambs, primarily due to enhanced digestive and absorptive utilization of fiber (100). Additionally, when switching from a high to a low concentration diet, the addition of yeast culture can improve feed efficiency without affecting growth, which can be attributed to the yeast culture making the diet more digestible, reducing fecal output, and enhancing feed absorption and utilization (101). Ovinge et al. (102) highlighted that live yeast can improve the digestion, absorption, and utilization rates of dry matter, crude protein, crude fat, and fiber. The introduction of yeast and yeast products into the industry has significantly improved breeding efficiency, facilitated green breeding strategies, alleviated the food crisis, and made substantial contributions to the development of the human and animal husbandry industries. The effects of yeast culture on ruminant production performance and digestion and absorption capacity (100, 103–105) are summarized in Table 3.

**Table 3:** Effects of yeast culture on ruminant’s production performance and digestion and absorption capacity.

Animal	Conclusion	References
Nili-Ravi buffaloes	Yeast cultures induced significantly more growth and production performance than did live yeasts.	(103)
Lambs	Yeast culture products can be added to the pelleted total mixed ration to improve the growth performance of lambs.	(100)
Lambs	Yeast culture improves the growth performance of lambs.	(104)
Ruminants	The addition of yeast can increase the feed intake and growth rate of young ruminants, and increase the milk yield and milk fat rate of dairy cows	(105)

## 5 Discussion

**Figure 1:**
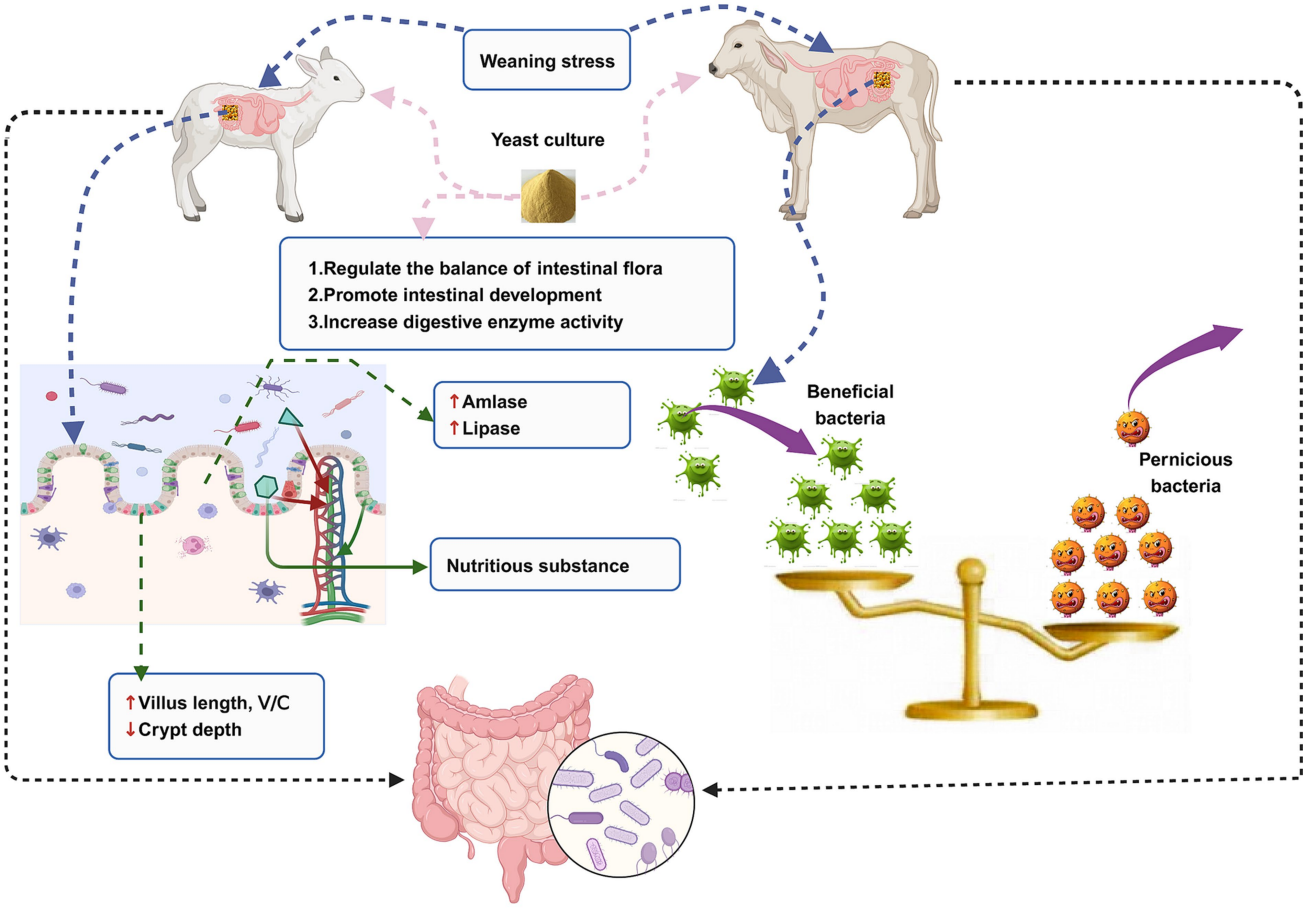
An illustration of the relationship between intestinal microorganisms, intestinal health, weaning stress, and yeast culture in the young ruminants.

The animal intestine is colonized by a large number of microorganisms that play important roles in maintaining host nutrition metabolism, intestinal tissue development, and intestinal immune function. Ruminants at a young age go through a period of rapid establishment of ruminant intestinal tissue, physiological function, intestinal immune system, and microbiota. At this stage, they are susceptible to weaning stress, which destroys the balance of intestinal flora and the development of intestinal tissue morphology, leading to intestinal disease and consequently serious economic losses to the livestock industry. With the continuous development of science and technology, it has been discovered that the incorporation of yeast culture to ruminant diet could significantly alleviate the damage caused by weaning stress to the intestinal health of young ruminants. This is an effective strategy to promote the balance of intestinal flora, the development of intestinal tissue, and the establishment of the mucosal immune system (Figure 1). This study provides a scientific basis for green and healthy breeding, a foundation for reducing animal diseases and ensuring animal health, and a theoretical basis for the application of yeast culture to the diet of young ruminants.

## Author contributions

SL: Writing – original draft. LY: Writing – review & editing. YZ: Writing – review & editing. HC: Writing – review & editing. XuL: Writing – review & editing. ZX: Writing – review & editing. RD: Writing – review & editing. XiL: Writing – review & editing. JM: Writing – review & editing. DL: Writing – review & editing.
